# Comparison of Laser Milling Performance against Difficult-To-Cut Alloys: Parametric Significance, Modeling and Optimization for Targeted Material Removal

**DOI:** 10.3390/ma12101674

**Published:** 2019-05-23

**Authors:** Naveed Ahmed, Madiha Rafaqat, Kashif Ishfaq, Ateekh Ur Rehman, Adeel Hassan, Usama Umer, Adham Ezzat Ragab, Ayoub Al-Zabidi

**Affiliations:** 1 Department of Industrial and Manufacturing Engineering, University of Engineering and Technology, Lahore- 54890, Pakistan; Madiharafaqat@yahoo.com (M.R.); kashifishfaq1@gmail.com (K.I.); 2 Department of Industrial Engineering, College of Engineering, King Saud University, Riyadh-11421, Saudi Arabia; arehman@ksu.edu.sa (A.U.R.); aragab@ksu.edu.sa (A.E.R.); 3 Department of Mechanical Engineering, University of Lahore, Islamabad 44000, Pakistan; adeel.hassan@me.uol.edu.pk; 4 Advanced Manufacturing Institute, King Saud University, Riyadh-11421, Saudi Arabia; uumer@ksu.edu.sa; 5 Graduate Student, Department of Industrial Engineering, College of Engineering, King Saud University, Riyadh-11421, Saudi Arabia; 439106932@student.ksu.edu.sa or alzabidi_8@hotmail.com

**Keywords:** laser milling, titanium alloy, nickel alloy, aluminum alloy, material removal rate, variation, MRR_%_, MRR_act_, models, optimality

## Abstract

During laser milling, the objective is not always to maximize the material removal rate (MRR). Milling of new material with targeted MRR is challenging without prior knowledge and established sets of laser parameters. The laser milling performance has been evaluated for three important aerospace alloys, i.e., titanium alloy, nickel alloy and aluminum alloy using the response surface method experimental plan (54 experiments for each alloy). Parametric effects of five important laser parameters, statistical analysis (main effects, interaction effects, strength and direction of effects), mathematical modeling and optimality search is conducted for the said alloys. Under the non-optimized laser parameters, the actual MRR significantly varies from the targeted MRR. Variation in the aluminum alloy is at the top as compared to the other two alloys. Among other significant terms, three terms have the largest effect on MRR in the case of TiA, two terms in the case of NiA, and five terms in the case of AlA. Under the optimized sets of laser parameters, the actual material removal highly close to the desired level (100%) can be achieved with minimum variation in all the three alloys. Mathematical models proposed here have the capability to well predict material removal prior to the actual machining of Ti6Al4V, Inconel 718 and AA 2024.

## 1. Introduction

Among the list of materials used in the aerospace and automobile sector, titanium alloy (Ti6Al4V), nickel alloy (Inconel 718) and aluminum alloy (AA 2024) have frequent use [1]. These alloys are very well known for their high resistance to corrosion and sustainability in aggressive environments [2]. In the material development, research is continuously being carried out to further improve the mechanical properties such as good strength and resistance against corrosion when the material is assumed to be used under high temperature conditions. The said alloys are also highlighted in the category of difficult-to-cut materials in the field of machining. Conventional machining processes face lots of difficulties especially in terms of high cutting forces and frequent tool wear [3]. The challenges during conventional machining are being overcome with thermal assistance in conventional processes [4]. Thermal assisted machining like laser assisted machining (LAM) is considered as the widely employed alternate to deal with difficult-to-machine materials such as super-alloys [5]. Alternatively, to deal with the said alloys, non-conventional machining practices are therefore employed such as ultrasonic machining, ultrasonic assisted machining, electrical discharge machining (EDM), electrochemical machining, and laser machining [6,7,8,9,10,11]. Although the application domain of these processes varies but they offer less difficulty because of the absence of many conventional factors such as cutting forces, tool wear and tool failure, etc. However, the commonality of this stream of processes is their low machining rate. That is the reason why, the majority of the research is being carried out to accelerate the material removal rate. Research towards achieving a high MRR during the EDM of Inconel 718 can be seen in [12]. 

Laser machining is one of the competent processes to machine a huge range of materials. It has many variants and different terms are used such as laser beam machining (LBM), laser cutting, laser ablation, laser beam drilling and laser milling. Arrizubieta et al. [13] proposed a combined process of laser deposition, laser milling and laser polishing to manufacture the part with completeness in each aspect. The process has been validated on Inconel 718. Laser milling is capable of developing 2D as well as 3D shaped features ranging from macro to microscale. Nanostructures can also be produced through the laser machining process [14,15]. Torres et al. [16] produced dimple-like textures through the Nd:YVO_4_ laser in the aluminum alloy (AA 2024) and texture quality is evaluated against laser parameters. Likewise, in [17] the picosecond laser is employed to study the surface textures produced in AA 2024. Microchannels, microcavities and micro and nanostructures are the typical outcomes of laser milling, which are directly linked up with applications in micromolding, heat sinks, and biomedical implants [18,19]. The penetration depth in AA 2024 after laser peening is studied in [20] and the effect of laser parameters are investigated on the machining behavior. Networks of microchannels are produced in the metallic foil by Shen et al. [21].

The laser milling process consists of numerous parameters such as the current intensity, laser fluence, pulse duration, repetition rate, scan passes, scan strategies, layer thickness, spot size, beam focus, spot overlap, wavelength and others [22]. This long list of parameters acting together makes the process nature multifarious. The rate of material removal and the consequent dimensional accuracy exponentially varies with a minute change in process parameters. That is the reason why, the optimized sets of parameters for predefined objectives are really desired during laser milling [23]. Pulse frequency has a great contribution in laser processes and is reported as one of the most significant parameters [24]. Schille et al. [25] also reported that a high pulse frequency is more favorable to get an accelerated MRR. Yang et al. [26] measured the oxidation layer thickness on Ti6Al4V developed during nanosecond laser milling. Among the parametric effects, a low scan speed along with a congested hatch distance created a thicker layer. Another research [27] reports on the aggressiveness of laser milling under the conditions of low pulse repetition rate, slow scan speed and high current intensity. As a result, a high MRR is noticed. Important parameters affecting MRR during the Ytterbium laser machining of the aluminum composite are identified in [28]. Laser power, pulse frequency and pulse width are found to be the significant variables. Mathematical models are also developed to achieve a maximum MRR and lowest taper. A similar study is done to achieve a high MRR with minimum taper [29]. It has been stated by Hussain et al. [30] that a precise correlation between input variables and output responses is very essential and difficult to develop during laser machining. In this connection, Yu et al. [31] produced micro-grooves in Ti6Al4V through picosecond laser and proposed a correlation between laser parameters and feature geometry. The laser milling performance is also considered as the function of the substrate’s thermal and physical properties. The influence of the laser parameters on the channel’s geometry has been studied by the researchers of [32]. They have analyzed the effect of laser power and scanning speed on the milling depth and both of these parameters are rated as significant variables. The pulse overlap between consecutive laser scans has also been reported as the contributing factor towards the morphology of the laser machined profile [33]. It is recommended that the right choice of laser parameters is essential to get the desired depth during laser milling of polymethylemethacrylate (PMMA). Seeking an optimized combination of parameters and mathematical models for micromachining can be witnessed in [34].

A common practice during the optimization of laser process parameters with respect to the milling performance is towards the material removal rate, surface roughness, and geometry of milled profile. With respect to optimization for the material removal rate, the goal is set to achieve either the highest MRR or optimal MRR. Optimization of laser parameters is reported in [35] to get the optimal geometry of microchannels. The goals were to obtain a maximum MRR and minimum surface roughness. While practicing the Nd:YAG laser milling, Teixidor et al. [36] proposed an optimal set of process parameters against the set goals of achieving an optimal milling depth and volume of microcavities. The maximum material removal with minimum surface quality during laser milling of ceramics are researched by Umer et al. [37].

From the literature, it can be stated that the material removal plays a pivotal role in laser beam processes. Especially, during laser milling the precision and accuracy of the milled feature primarily depends on the material removal rate, which should be precisely controlled in a layer-by-layer fashion. Thus, it cannot be said that maximizing MRR will always resolve the issues of laser milling performance in terms of the feature’s dimensional accuracy; the milling depth in particular. If the material removal rate were excessive compared to the anticipated rate then the milling depths would be exceptionally high. Higher milling depths during the pulsed laser system are reported as the cause of surface changes ultimately leading towards the unevenness surface generation [38]. Therefore, in this research laser milling has been performed on three very well-known difficult-to-cut alloys (Ti6Al4V, Inconel 718, and AA 2024). The performance of laser micromilling is compared with respect to the said alloys in terms of MRR. The theoretically calculated MRR and experimental MRR evaluated together and the percentage material removal rate (MRR_%_) is taken as the common parameter of comparison. Five important laser parameters are considered as the input variables to speculate their influence and contribution towards the material removal. For each alloy, significant variable terms are identified in their linear, quadratic and interaction effects. Moreover, the strength and direction of each parametric effect on each alloy are evaluated. Since, in this research the main target was set to seek those process conditions which are promising to gain the desired and targeted amount of the material removal, therefore, the MRR_%_ is set to equate at 100% value. In this connection, the optimized sets of laser parameters having the capability to result into MRR_%_ highly close to 100% for each of the three alloys, i.e., titanium alloy (TiA), nickel alloy (NiA) and aluminum alloy (AlA) are proposed. Mathematical models that the practitioners can confidently use to predict the material removal before doing actual milling are also developed and validated, since the R-square value of each model is well above 90%.

## 2. Experimental Details

Alloys of titanium, nickel and aluminum have become common materials in various industries including the aerospace sector. Milling of these three alloys is performed through the Nd:YAG laser machining (DMG Mori Seiki Co., Nagoya, Japan). Details of research materials, setup, and design of experiments are provided in the subsequent sections.

### 2.1. Research Materials

Three important aerospace alloys are taken as the research materials, which include titanium alloy (TiA), nickel alloy (NiA) and aluminum alloy (AlA) with grades Ti6Al4V, Inconel 718, and AA2024, respectively. Due to their extensive use in the industry in various forms, milling is frequently required. Therefore, the laser milling performance has been investigated for the said materials in order to understand the machining behavior of each material when subjected to laser irradiations. The elemental composition in wt% is presented in Table 1. Since the performance of milling directly relates with the substrate properties (e.g., absorptivity, reflectively, and melting point etc.) [32], therefore important properties of the research materials are provided in Table 2. Work samples with similar geometrical dimensions are chosen. Each specimen is a square cross-sectional ingot consisting of 25 mm length, 6 mm width and 6 mm breadth. Flatness of the work surface is imperative in order to have an equal reference for laser spot focusing. Thus, each specimen is surface ground to maintain a flat surface with uniform roughness.

### 2.2. Setup, Variable Selection and Design of Experiments

**Figure 1:**
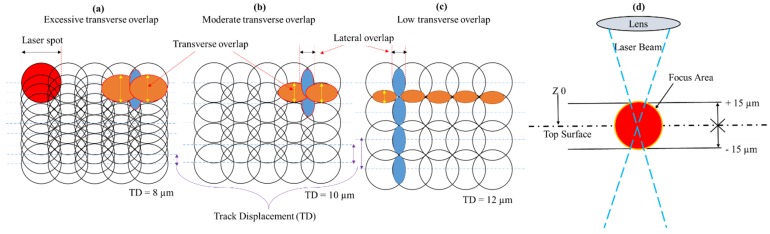
Schematic of track displacement (TD) and overlapping of laser spot: (**a**) Excessive transverse overlap; (**b**) moderate transverse overlap; (**c**) low transverse overlap; and (**d**) focus of laser spot.

**Figure 2:**
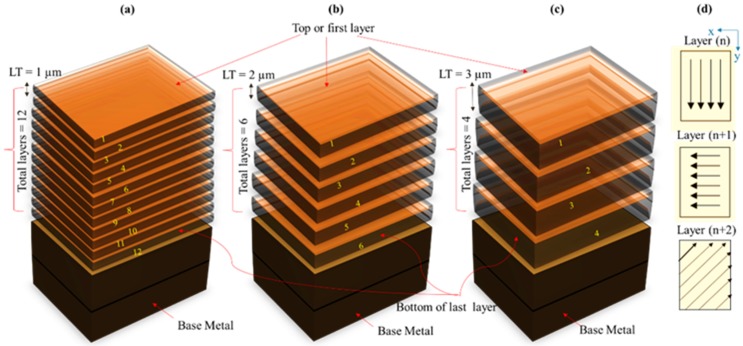
Schematic of layer thickness (LT) and number of layers: (**a**) 12 layers when LT = 1 µm; (**b**) six layers when LT = 2 µm; (**c**) four layers when LT = 3 µm; and (**d**) scan directions.

In this research, the Q-switched Nd:YAG laser machine (model: Lasertec 40) has been used to perform the experiments. It has the capability to produce the laser beam in the Gaussian mode with 30W power, 1064 µm wavelength, 10 µs pulse duration and 20 µm spot size. Rectangular cross-sectional slots are milled in each of the said alloys. The length, width and depth of the slots are 5 mm, 3 mm and 12 µm, respectively. The width of 3 mm and depth of 12 µm results into the rectangular cross-section of the milled slot. Laser intensity and scan speed are considered as the important parameters while commencing laser milling in any material. So, prior to executing the design of experiments, various laser parameters are tested to identify the workable range of different parameters especially laser intensity. Thus, the ranges of parameters are decided based on the trials and the manufacturer’s guided scheme. Five parameters are taken as variables, i.e., laser intensity (I), pulse frequency (f), scan speed (V), track displacement (TD) and layer thickness (LT). The milling performance is evaluated in terms of material removal rates (MRR) corresponding to the said alloys. Details of variables, their levels and response indicators are provided in Table 3. According to the feature profile, the laser beam starts its travel along predefined tracks. During travel along the first line, the preceding laser spot overlaps the forthcoming spot in one direction. Likewise, overlapping occurs for the second line. In this way, two types of overlapping came into existence, i.e., lateral overlapping and transverse overlapping as labeled in Figure 1. Due to the high density of spot overlap the scanning takes a larger time to complete the scan cycle. High density overlapping also generates high laser energy density per unit area and excessive melting may be resulted. Therefore, keeping in view the contribution of overlapping a parameter named track displacement (TD) is considered as a variable factor. Three levels of TD are taken, i.e., 8 µm, 10 µm and 12 µm. The low value of TD indicates high density overlapping and the high level means low density overlapping. Hence, three cases are nominated as excessive, moderate and low overlapping as depicted in Figure 1a–c. Different regions of the laser beam in the Gaussian mode have varying levels of energy with the highest level of energy at focus point. The focal length is adjusted in such a way that the focus of the laser beam remains at the top surface of the work piece as schematically represented in Figure 1d. After completing the scan cycle, the material is removed and the fresh surface layer is exposed to the incoming beam. To keep the focus on top of the fresh layer, the laser spot needs to be re-focused. This re-focusing is based on the thickness of the removed layer, which is termed as layer thickness (LT) and is one of the current research variables. Three levels of LT are considered, i.e., LT of 1 µm, 2 µm, and 3 µm, which means that after every scanning cycle per layer the focal distance is adjusted accordingly. For example, LT of 3 µm indicates that for each fresh layer the focal length would be adjusted (through the Galvano head) with an increment of 3 µm. The amount of layer thickness also determines the total number of scan cycles. For example, to accomplish the milling depth of 12 µm with 3 µm layer thickness, the corresponding number of scan cycles would be four. Similarly, six cycles with 2 µm LT and 12 cycles with 1 µm LT are required to complete the scanning cycles for 12 µm depth. The whole concept of layer thickness can be envisioned from Figure 2. There were three scan strategies or scan directions to choose for milling as depicted in Figure 2d. The random mode scan strategy is adopted for each experiment. 

In order to understand the process behavior and contribution of laser parameters on the material removal the response surface method of the experimental design is selected. As per design, 54 experimental runs are performed with each of the three alloys (TiA, NiA and AlA). In total, 162 experiments are conducted to investigate the milling performance of the three alloys. 

### 2.3. Measurements and Calculations

After each experiment, the measurement of the milling depth is carried out at three different locations of the milled surface with the help of the measurement probe of *Lasertec 40*. The average depth is taken as the input for the material removal rate (MRR) calculation. The machining time consumed in completing the predefined scan cycles and depth is recorded in each experiment. Theoretically, the machined volume should be equal to the volume of the designed rectangular shaped slot (depth×width×length) but in actual it varies from the designed volume. Thus, based on this fact, the theoretical material removal (MRR_th_) and actual experimental material removal (MRR_act_) are determined using Equations (1) and (2), respectively. Due to the influence of varying levels of parameters, the experimental values of MRR vary from the theoretical MRR. Each combination of variables generates different levels of energy density. If the energy available per unit area is insufficient to melt the desired thickness of the substrate layer the actual machined depth or volume would be less than the desired amount of the depth or volume. And if the combination of variables provides excessive energy density per unit area, the resulting depth could be undesirably higher than the anticipated depth. This variation in the material removal further varies from material-to-material because of different properties. For example, with respect to the three alloys, the thermal conductivity of NiA (10.6–29.6 W/m °C) is less as compared to TiA and AlA (32.74 and 164–220 W/m °C) which means that heat accumulation underneath the laser spot is more in the case of NiA. Similarly, the emissivity difference with respect to the three alloys indicates that under the same parametric conditions the effect of the laser beam would obviously be different. The low dynamic viscosity of AlA (1.3 × 10^−3^ Ns/m^2^) allows the ablated debris to be removed more efficiently as compared to TiA with the high dynamic viscosity (5.20 × 10^−3^ Ns/m^2^). The result would be a high MRR with greater milling depth in the case of AlA as compared to NiA. Similar cases are with other properties such as density, melting points, and absorptivity etc., the consequential effect is on the ablation depth or material removal rate due to which huge variations are observed between the actual MRR and theoretical MRR.

**Figure 3:**
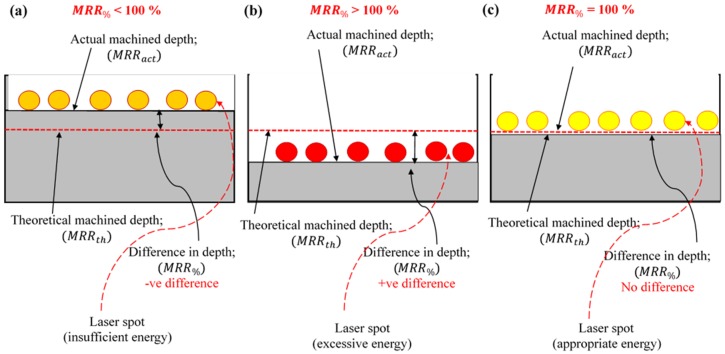
(**a**) MRR_%_ < 100% with −ve difference; (**b**) MRR_%_ > 100% with +ve difference; and (**c**) MRR_%_ = 100% with no difference.

Therefore, the material removal in terms of the percentage (MRR_%_) is introduced in this research to simplify the understanding of the material removal variation. The MRR_%_ is calculated using Equation (3). Based on this equation, if the MRR_%_ is less than 100% it means that the experimental depth or volume is less than the desired depth or volume and consequently the experimental MRR would be less than the theoretical MRR. The difference between bot MRRs is −ve in this case. If this is the case, then the accuracy of the final milled feature is compromised. Similar is the instance when the experimental depth is higher than the designed depth of feature, the case of +ve difference between the MRR_th_ and MRR_act_. Hence, if MRR_%_ > 100%, it indicates an oversized milled feature. High dimensional accuracy in laser milling can be ensured if the MRR_%_ is exactly equal to 100%, which means that there is no difference between the designed and actual depth. These three cases of MRR variations are schematically illustrated in Figure 3.

(1) $$Theoratical\;Material\;Removal\;Rate=MRR_{th}=\frac{{\left(Depth\times width\times length\right)}_{theoratical}}{\left(Machining\;time\right)}$$

(2) $$Actual\;Material\;Removal\;Rate=MRR_{act}=\frac{{\left(Depth\times width\times length\right)}_{actual}}{\left(Machining\;time\right)}$$

(3) $$Percent\;Material\;Removal\;Rate=MRR_{\%}=\frac{MRR_{act}}{MRR_{th}}\times 100$$

## 3. Results and Discussion

**Figure 4:**
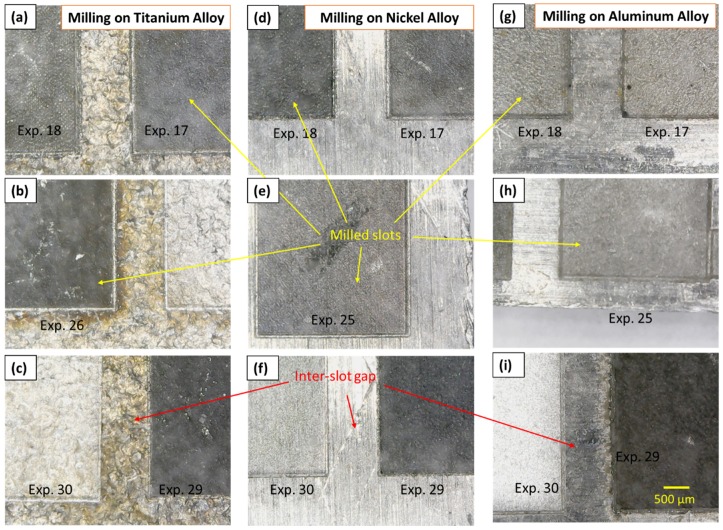
Microscopic images of selected milled slots: (**a**–**c**) Milling on TiA; (**d**–**f**) milling on NiA; and (**g**–**i**) milling on AlA.

Laser milling of titanium, nickel and aluminum alloys has been performed under the response surface method (RSM) design of experiments. The theoretical material removal rate has been calculated for each experimental run, which is the function of the designed volume and machining time. Furthermore, machining time is the function of layer thickness, scan speed and track displacement and the number of scanning passes. Thus, the theoretical MRR has initially been defined as a base line for comparing the actual MRR achieved for each of the three alloys (TiA, NiA and AlA). Certain experimental results of percentage material removals are presented in Table 4. Selected milled slots in each of the three alloys are presented in Figure 4. From the results, first the identification of parametric trends is carried out and second the effect of each of the five variables on the MRR associated with TiA, NiA and AlA are evaluated through the main effects and two-way interaction effects plots. Segregation of control variables into significant and insignificant variables is done through the analysis of variance. After catching the significant terms, the strength as well as the direction of effects is determined through Pareto and standardized normal plots. Mathematical models for MRR_%_ corresponding to each of the three alloys are developed and optimal parameters are sought. Verification of models and optimality search is finally established.

### 3.1. Identification of Trends

**Figure 5:**
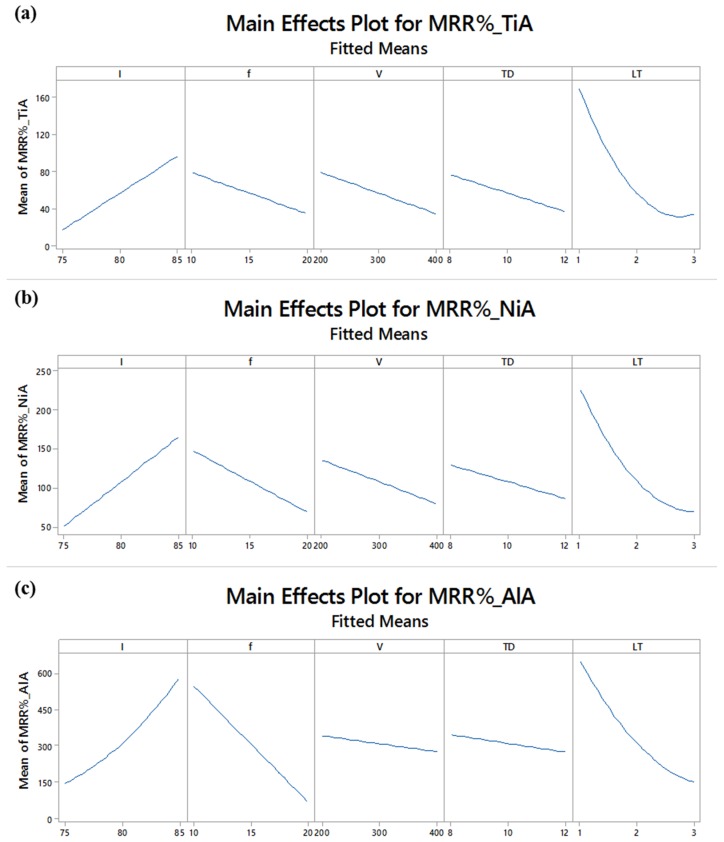
Identification of parametric trends w.r.t percentage material removal rate (MRR_%_): (**a**) Trends for MRR_%__TiA; (**b**) trends for MRR_%__NiA; and (**c**) trends for MRR_%__AlA.

**Figure 6:**
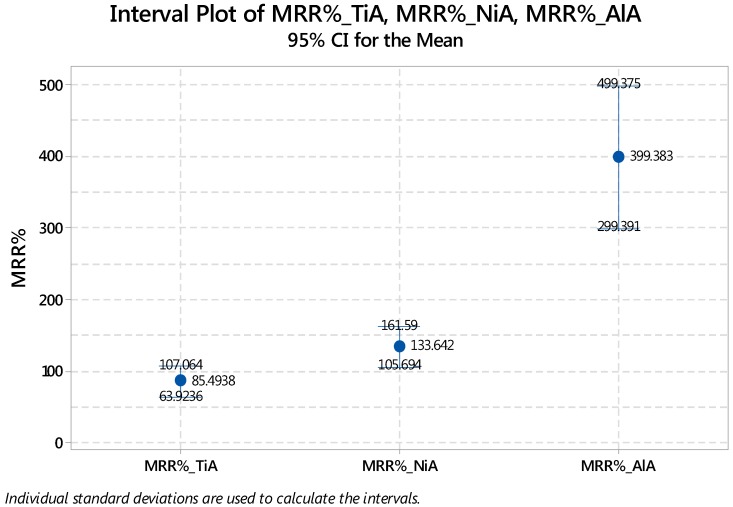
Interval plot of MRR_%_ associated with TiA, NiA and AlA.

Main effect plots associated with substrate materials are shown in Figure 5. It can be seen that with the increase in the lamp current intensity (I), the percentage material removal also increases. As the current intensity is increased the strength of the laser energy available per unit time is increased and the melting rate gets increased. The result is deep milling due to which the variation of the actual material removal with respect to theoretical values is more and therefore the percentage MRR gets increased. The effect of the other four variables, i.e., f, V, TD and LT are found to be inversely proportional to the material removal. The individual or simultaneous increase in pulse frequency, scan speed, track displacement and layer thickness causes the percentage of the material removal to be on the lower side. This relationship of control variables remains the same for each of the tested alloys. However, it can also be witnessed that the slope of the line in each graphical subset is different for different materials. For example, in the subsets corresponding to the pulse frequency, the line is steeper in the case of the aluminum alloy as compared to the same lines against the titanium as well as the nickel alloy. A clear difference with respect to the y-axis scales can also be noticed which indicates that a huge variation in the material removal exists. Although the trend lines are similar for each alloy but the mean values of their starting and ending points soundly differs. The interval plot shown in Figure 6 indicates that the interval for MRR_%_ corresponding to TiA is 64–107%, whereas the intervals associated with NiA and AlA are 105–161% and 300–500%.

**Figure 7:**
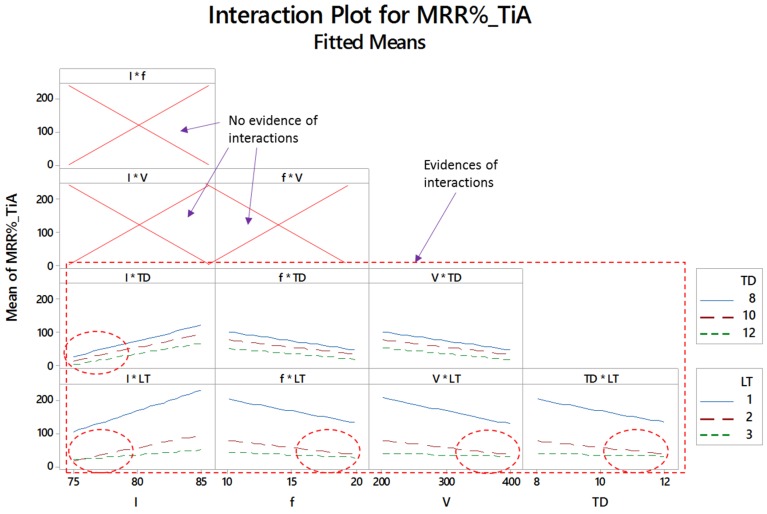
Interaction plots for percentage material removal rate for titanium alloy (MRR_%__TiA).

**Figure 8:**
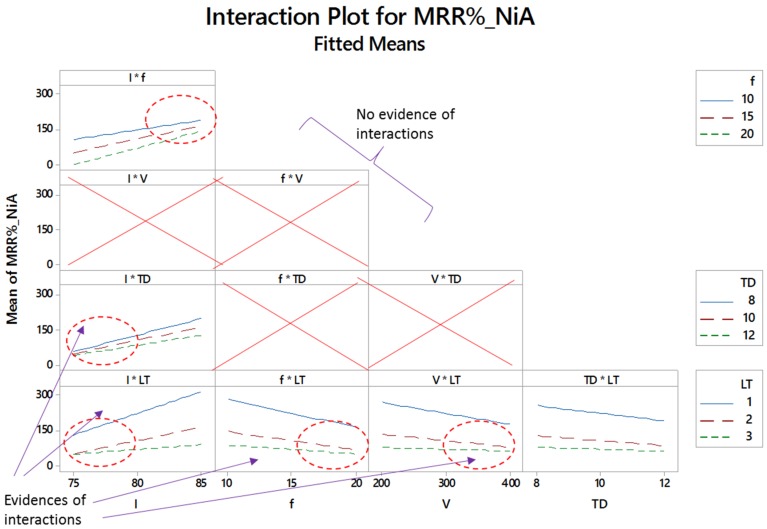
Interaction plots for percentage material removal rate for nickel alloy (MRR_%__NiA).

**Figure 9:**
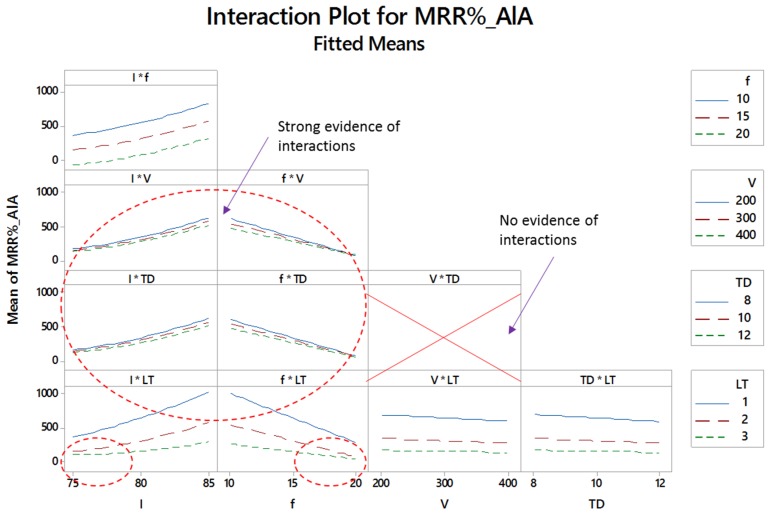
Interaction plots for percentage material removal rate for aluminum alloy (MRR_%__AlA).

Figure 7, Figure 8 and Figure 9 show the two-way interaction plots associated with the titanium, nickel and aluminum alloy, respectively. The higher the lines intersect, the higher the interaction effect. Some of the lines are parallel to each other indicating that there is no existence of the interaction effect. However, if the lines are non-parallel then the corresponding variables collaborate with each other and a combined effect on the material removal is generated. The number of intersections is different for all the three alloys. In some cases, no evidence of the interaction effect has been noticed. The subsets presented in Figure 7, Figure 8 and Figure 9 indicate that the effects of control parameters in terms of the two-way interaction is present for each of the titanium, nickel and aluminum alloy but not for all combinations of parameters. Further details of interaction effects and the number of interaction terms are discussed in the statistical analysis. 

### 3.2. Parametric Effects and Substrate Comparison

**Figure 10:**
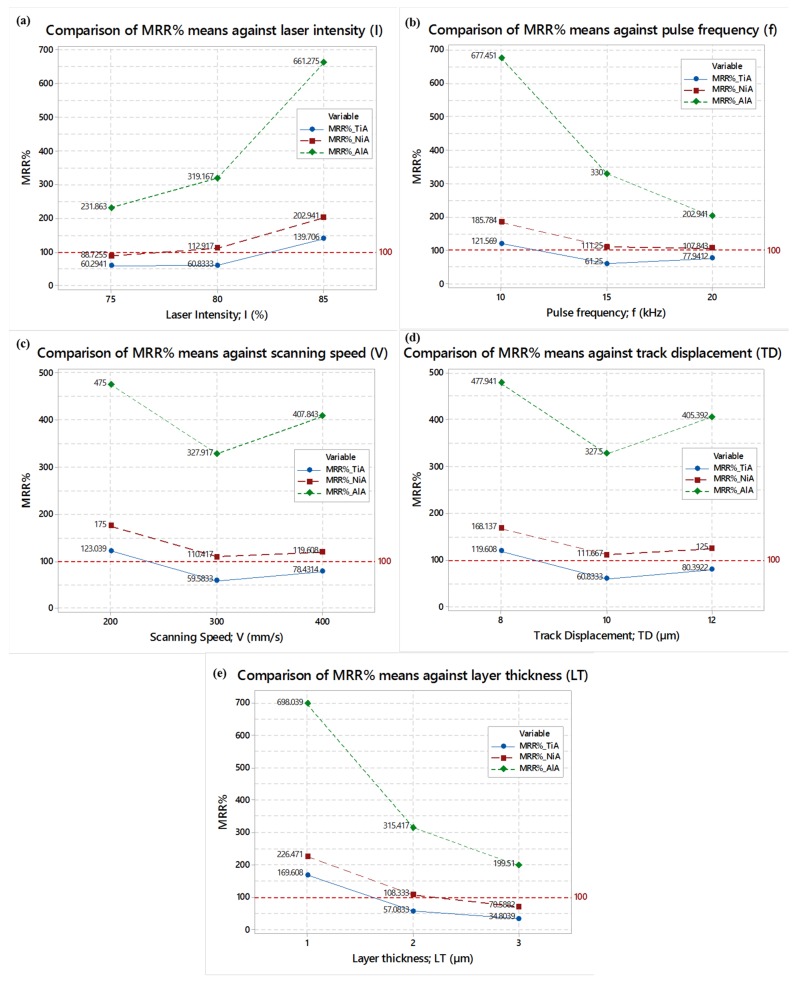
Comparison of laser milling parametric effects on MRR_%_: (**a**) Comparison against laser intensity; (**b**) comparison against pulse frequency; (**c**) comparison against scan speed; (**d**) comparison against track displacement; and (**e**) comparison against layer thickness.

The effects of each of the five laser parameters on the material removal of Ti6Al4V, Inconel 718 and AA 2024 are studied in the form of comparison among the three alloys. The results are shown in Figure 10. A reference line lying at 100% MRR is marked to compare the performance of the substrate material. From Figure 10a, it can be observed that irrespective of the substrate material, the higher level of laser intensity imparts the high percentage of material removal. TiA and NiA behave similar to each other and their MRR remains close to the reference line. However, at each intensity level, the material removal of AlA is well above than the desired MRR_%_ (100%), which is possibly due to the low melting point of AA 2024 as compared to the other two alloys (see Table 2). Similar kind of observations (but in reverse order of trend lines) can be witnessed from Figure 10b, wherein the effect of the pulse frequency is presented. The high level of pulse frequency is found to be more suitable in order to keep MRR_%_ close to the reference line (MRR_%_ = 100%) for the three alloys. At low frequency (10 kHz), the MRR variation is extremely high in the case of AlA. However, at this frequency TiA and NiA both results into more than 100% MRR which is even undesirable. Figure 10c indicates that the laser scan speed of 300 mm/s can yield the MRR approaching towards the reference line. Although, MRR associated with AlA is well above the desired value (100%) but in comparison to the other two extremes of scan speed, the middle value allows the material removal to be closer to the reference line. Exact similar observations are recorded in the case of the track displacement (TD) effect. At a TD of 10 µm, all the three alloys exhibit less variation of MRR with respect to the referenced value. The effect of the layer thickness (LT) on MRR is very much similar to the effect of the pulse frequency. The sub-graph of Figure 10e depicts that a layer thickness of 3 µm could allow the laser beam to melt the material in such a way that the resulting variation in MRR would be less for each of the three alloys.

During this comparison, a common observation has been noticed. In the case of the titanium alloy and nickel alloy, the difference of MRR from the theoretical MRR is relatively low. However, in the case of the aluminum alloy the said difference is extraordinarily high which is mainly due to the prominent difference of the physical and thermal properties of AlA as compared to those of the titanium and nickel alloy, as presented in Table 2.

### 3.3. Statistical Analysis

In order to quantify the laser milling performance against the titanium alloy, TiA (Ti6Al4V), nickel alloy; NiA (Inconel 718), and aluminum alloy; AlA (AA 2024), statistical analysis is performed. It included analysis of the variance, analysis for strength of effects and analysis of direction of effects. 

#### 3.3.1. Analysis of Variance

Analysis of variance (ANOVA) for each of the three alloys has been categorically performed to identify and segregate the control variables into significant and non-significant variables. The selection criteria of a 95% confidence level is set to qualify three categories of terms, i.e., linear terms, square terms, and two-way interaction terms. A total of 13 numbers of terms are found to be significantly contributing towards MRR_%_ in the case of TiA. It includes five linear terms, one square term and seven interaction terms. P-values of these terms are found to be either 0.00 or less than 0.05. In the case of NiA, 12 terms are found to be significant including five linear terms, one square term and six interaction terms. Since during parametric effects it is found that the behavior of AlA is soundly different than the other two alloys and the difference between MRR_%_AlA_ and MRR_%_th_ is high, therefore the same evidence has been noticed through the ANOVA results of AlA. A total of 16 terms are qualified as significant contributing terms in the case of AlA, which include five linear terms, two terms in square format, and nine terms with two-way interactions. Summary of the ANOVA results for each of the three alloys is presented in Table 5 (left half of Table 5).

#### 3.3.2. Analysis for Strength and Direction of Effects

After performing ANOVA tests, the strength of effects as well as the direction of effects are analyzed. Based on the strength of effects, terms are classified into three categories, i.e., top most significant terms, terms with largest effects, and terms with moderate effects. In order to categorize the effects in terms of their direction of effects, each of the strength categories is further filtered out into two classes i.e., terms with +ve effects and terms with –ve effects.

**Figure 11:**
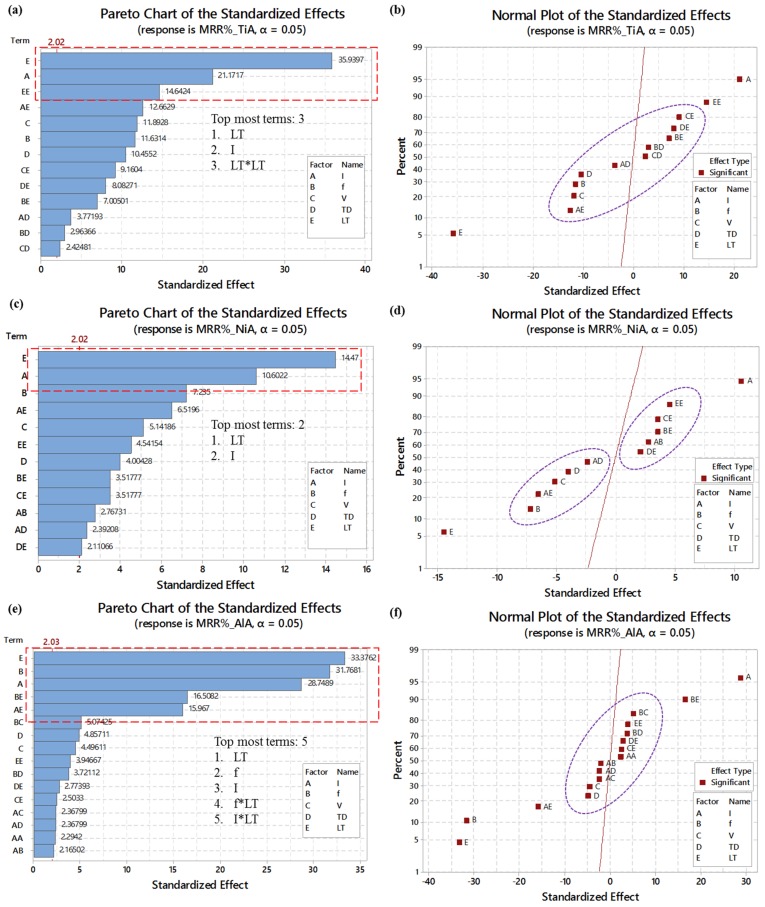
Strength of effects and direction of effects: (**a**) and (**b**) for TiA; (**c**) and (**d**) for NiA; and (**e**) and (**f**) for AlA.

Figure 11a,b shows the strength and direction of effects for MRR_%_ in the case of TiA. Out of 13 significant terms, three terms are found to be the most significant including layer thickness (LT), laser intensity (I) and square of layer thickness (LT^2^) as highlighted by the red-dashed rectangular callout. LT occupies the top place in the chart and can be mentioned as the 1st ranked term for TiA. Two of these terms (I and LT^2^) have a positive impact on MRR whereas LT has a negative impact. The remaining nine terms are found to be uniformly distributed around the reference line as shown in Figure 11b, which indicates that their impact, although significant, is comparatively moderate in strength. For full details about the direction of effects, the right half of Table 5 can be consulted.

In the case of nickel alloy (NiA), two terms are filtered as the most significant terms (see Figure 11c) including layer thickness (LT) at 1st rank and laser intensity (I) at 2nd rank. The directions of both of these effects are –ve and +ve, respectively. However, the remaining 10 terms are uniformly distributed around the reference line (see Figure 11d) five out of which have a positive effect (terms lying at the right side of the reference line) whereas five terms have a negative effect (left side terms). For full details, see the right half of Table 5.

On the other end, the strength of the effect on MRR_%_ associated with aluminum alloy (AlA), five terms out of 16 significant terms are experienced as the most strengthening terms as highlighted by the red-dashed rectangular callout in Figure 11e. The list of most significant terms for AlA include LT, f, I, f*LT, and I*LT. Layer thickness (LT) is again found to be at the top most position of the chart linked with MRR_%_ of AlA, so LT is at 1st rank for AlA as well. The direction of effects of these terms can be observed from Figure 11f. The remaining 11 terms are clustered around the reference line, which indicates that their impact on MRR, although significant, is relatively moderate. Full details about terms with the largest effect, moderate effect, +ve and –ve effect can be grasped from the right half of Table 5.

### 3.4. Modeling and Optimality Search

Statistical analysis performed over the results help to identify the significance of variables, their strength and direction of effects. Experimental results revealed that there is huge variation in the actual material removal rate (MRR_act_) with reference to the theoretical material removal (MRR_th_), as a result the percentage material removal (MRR_%_) is far away from the desired value (MRR_%_ = 100%). Descriptive statistics are presented in Table 6 as a reference. It can be seen that the maximum value of MRR_%_ is entirely different for different materials. The maximum exhibited value of MRR_%_ for TiA, NiA and AlA is 383%, 500% and 1600%, respectively. Due to this enormous variation, response surface regression models are felt to be developed so that the resulting amount of the material removal can be predicted well before the actual machining. The models associated with MRR_%_ of TiA, NiA, and AlA are shown in Equations (4), (5), and (6), respectively. The stepwise procedure is adopted to develop the models and only those terms are included in each of the models, which are identified as the significant terms. Summary of the models developed is given in Table 7, wherein the values of R-square and R-square adjusted (>90%) indicates that the models development is well accurate. The prediction ability of each of the proposed model is checked though R-square predicted values, which are well above the qualifying criteria, i.e., greater than 80%.

(4) $$\begin{array}{ll} MRR_{\%}\left(TiA\right)= & -899+25.02\;\left(I\right)-15.51\;\left(f\right)-0.812\;\left(V\right)+17.3\;\left(TD\right)-28.1\;\left(LT\right)\\ & +45.12\;\left(LT^{2}\right)-0.729\;\left(I\times TD\right)-4.896\;\left(I\times LT\right)+0.573\;\left(f\times TD\right)\\ & +2.708\;\left(f\times LT\right)+0.0234\;\left(V\times TD\right)+0.177\;\left(V\times LT\right)\\ & +7.812\;\left(TD\times LT\right) \end{array}$$

(5) $$\begin{array}{ll} MRR_{\%}\left(NiA\right)= & -1313+29.96\left(I\right)-64.8\;\left(f\right)-0.668\;\left(V\right)+83.7\;\left(TD\right)+165\;\left(LT\right)\\ & +40.20\;\left(LT^{2}\right)+0.615\;\left(I\times f\right)-1.328\;\left(I\times TD\right)-7.24\;\left(I\times LT\right)\\ & +3.91\;\left(f\times LT\right)+0.1953\;\left(V\times LT\right)+5.86\;\left(TD\times LT\right) \end{array}$$

(6) $$\begin{array}{ll} MRR_{\%}\left(AlA\right)= & 6860-202\;\left(I\right)-97.0\;\left(f\right)+1.02\;\left(V\right)+63.4\;\left(TD\right)+813\;\left(LT\right)\\ & +2.083\;\left(I^{2}\right)+\;89.6\;\left(LT^{2}\right)-0.667\;\left(I\times f\right)-0.0365\;\left(I\times V\right)\\ & -1.823\;\left(I\times TD\right)-24.58\;\left(I\times LT\right)+0.0781\;\left(f\times V\right)\\ & +2.865\;\left(f\times TD\right)+25.42\;\left(f\times LT\right)+0.1927\;\left(V\times LT\right)\\ & +10.68\;\left(TD\times LT\right) \end{array}$$

**Figure 12:**
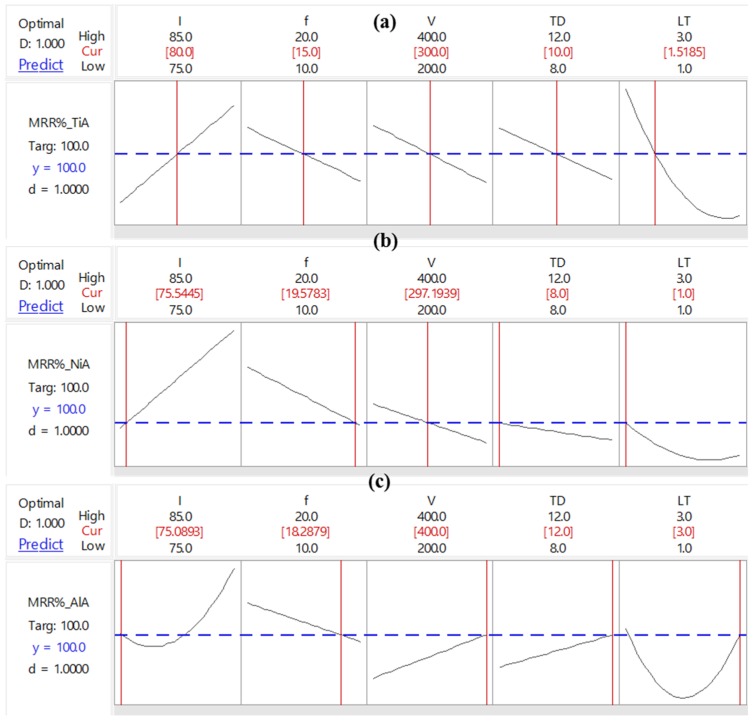
Optimization plots for MRR_%_ corresponding to: (**a**) TiA; (**b**) NiA and (**c**) AlA.

After regression modeling, the optimality search has been conducted to seek those sets of laser parameters, which are most suitable to result into an actual MRR_%_ close to the desired MRR_%_ that is set at 100%. The optimization goal for each of the three responses (MRR_%__TiA, MRR_%__NiA, and MRR_%__AlA) is set as “Target” to achieve a targeted value of MRR_%_ to be at 100% (Table 8). The optimization plot has been shown in Figure 12, whereas the summarized results of the optimization for each of the three alloys are presented in Table 9. Figure 12a represents the optimization results for the titanium alloy (TiA). The ted colored numeric values indicates the optimized settings of laser parameters whereas the blue dashed-line indicates the current value of MRR_%_ for TiA. Similar is the case for the nickel alloy and aluminum alloy as shown in Figure 12b,c, respectively. The desirability value of 1.00 is the indicator of the robustness of optimality search, which has been found in all three responses. It is worth noting that the confidence interval corresponding to TiA is relatively closer as compared to the confidence intervals associated with NiA and AlA. The confidence interval for the aluminum alloy is the widest among all three intervals. That is why, the standard error fit (SE fit) for AlA is higher (26.3) as compared to the SE fit of NiA and TiA (16.1 and 2.13). The SE fit for TiA is found to be very small (just 2.13) which is also evidenced in the highly congested confidence interval. 

### 3.5. Verification of Modeling and Optimality Search

In order to validate the mathematical models, three solutions for each alloy are predicted. The results are mentioned in Table 10. For each of the alloys, the first solution is corresponded to the optimized solution whereas for the remaining two solutions the parametric values of control variables are different. Consequent fitted values in terms of MRR_%_ are always found to be within the confidence intervals of corresponding substrate materials. Furthermore, utilizing the optimized settings of laser parameters each alloy was milled and the actual material removal was found to be highly close to the targeted value of 100% with an error below 5%. From the last column it can also be seen that the composite desirability against each predicted solution is well above 0.8, which is considered as a qualifying criteria for good prediction and optimality search. 

**Figure 13:**
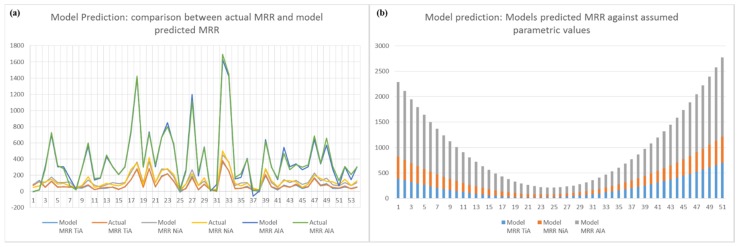
Validation of models prediction: (**a**) Comparison between actual and model predicted material removal rate (MRR); and (**b**) models predicted MRR against assumed set of laser parameters.

Model predicted MRR and actual experimental MRR values are compared to see the difference. Figure 13a shows that both values are very much close to each other. Additionally, the models are utilized to predict a wide range of solutions against an assumed set of parametric values. The assumed range of each of the five laser parameters (I, f, V, TD and LT) is selected in such a way that the assumed values are away from the minimum and maximum level of each variable tested in this research. The range of laser intensity is taken as 70–95% with an increment of 0.5%. As a result 51 solutions are predicted. Likewise, the range for the assumed set of pulse frequency is taken from 5 kHz to 30 kHz with an increment of 0.5 kHz. Scanning speed has been assumed starting from 100 mm/s and ending at 850 mm/s with an incremental difference of 15 mm/s. With an increment of 0.2 µm, the track displacement is chosen within 6–16 µm for the assumed set of 51 prediction solutions. A layer thickness ranging within 0.5–5.5 µm with an increment of 0.1 µm has been taken as the assumed values for prediction of 51 solutions. The model predicted results are presented in Figure 13b. It can be observed that very low assumed levels as well as extremely high assumed levels of each of the five laser parameters result into an aggressively high percentage of material removal rate for each of the three alloys (TiA, NiA, and AlA). However, at the moderate range of parameters the percentage of material removal rate is found to be at the lower side of MRR_%_. Thus, from Figure 13b, it can be evidenced that the proposed models (presented in Equations (4)–(6)) work well to predict the output response of laser milling of the titanium alloy, nickel alloy and aluminum alloy and so the laser milling operators can utilize the models to predict the material removal very quickly. An immense effort of searching for the appropriate parametric combinations and extensive need of trials can be avoided by the use of recommended optimized parameters as well as by the use of proposed models. 

## 4. Conclusions

Milling of three important aerospace alloys (TiA; Ti6Al4V, NiA; Inconel 718 and AlA; AA 2024) has been carried out through the Q-switched Nd:YAG pulsed laser. Performance of laser milling in terms of the percent material removal rate (MRR_%_) is evaluated through parametric effects of five important laser parameters and statistical analysis. In order to achieve the targeted and desired amount of the material removal rate for each of the said alloys, mathematical models are developed. Optimized sets of process parameters are sought to be capable of producing the exact milling volume based on 100% material removal rate. Experimental results obtained over the response surface methodological design of experiment, discussion, model prediction analysis, and optimality search lead to infer the following iconic conclusions from the present study:

1. Laser milling of the titanium, nickel and aluminum alloys can be performed through the Nd:YAG pulsed laser. However, the actual material removal rate (MRR_act_) doesn’t always remain under control if the objective is to target the material removal rate as per the desired level.
2. With respect to the three alloys under investigation, AlA always exhibits a larger variation in the material removal rate as compared to TiA and NiA when subjected to laser milling against the non-optimized laser parameter. The mean values of MRR_%_ are found to be approximately 85%, 133% and 400%, whereas the maximum values of MRR_%_ are found to be 383%, 500% and 1600% for TiA, NiA and AlA, respectively.
3. The main reason behind the variations in milling results against non-optimized parametric combinations is the differences among the thermo-physical properties of the said alloys. The leading properties include the thermal conductivity and emissivity of the substrate. High thermal conductivity and low emissivity of AlA could be the reasons behind the aggressive milling variation compared to the other two alloys.
4. Mismatch within the parametric values, especially at extreme levels of variables, allow the laser beam to interact for insufficient or a longer period of time causing an undesired amount of material removal. For example, high laser intensity along with low levels of scanning speed, layer thickness, and track displacement causes excessive melting per unit area and results into undesirable deeper cuts.
5. Various terms in their linear effects, squared effects and two-way interaction effects contribute towards the material removal.
  - For TiA, 13 terms are found to be significant including five linear terms (I, f, V, TD, LT), one square term (LT*LT) and seven interaction terms (I*TD, I*LT, f*TD, f*LT, V*TD, V*LT, and TD*LT).
  - For NiA, 12 terms are found to be significant including five linear terms (I, f, V, TD, LT), one square term (LT*LT) and six interaction terms (I*f, I*TD, I*LT, f*LT, V*LT, and TD*LT).
  - For AlA, 16 terms are found to be significant including five linear terms (I, f, V, TD, LT), two square terms (I*I and LT*LT) and seven interaction terms (I*f, I*V, I*TD, I*LT, f*V, f*TD, f*LT, V*LT, and TD*LT).
6. There are three most significant terms which have the largest effect on MRR_%_ for TiA including LT, I and LT*LT. For NiA, among 12 significant terms two terms (LT and I) have prominently the largest effect. In the case of AlA, five terms (LT, f, I, f*LT, and I*LT) are filtered out as the top most significant terms among a pool of 16 significant terms.
7. With respect to the strength of parametric effects, layer thickness (LT) is the common laser parameter, which is ranked at the 1st place for each of the three alloys.
8. The developed mathematical models (presented in Equations (4)–(6)) can be confidently utilized to predict MRR_%_ associated with TiA, NiA and AlA. Models predicted solutions are verified through actual experimentation as well as through assumed set of 51 parametric combinations.
9. Using the optimized sets of laser parameters, the actual MRR_%_ highly close to the desired level (MRR_%_ = 100%) can be achieved for each alloy, thus the variation in milling performance can be minimized irrespective to the substrate alloy subjected to laser irradiations.
  - For TiA, the optimized parameters are: Laser intensity (I) at 80%, pulse frequency (f) at 15 kHz, scan speed (V) at 300 mm/s, track displacement (TD) at 10 µm, and layer thickness (LT) at 1.51 µm.
  - For NiA, the optimized parameters are: Laser intensity (I) at 75.54%, pulse frequency (f) at 19.57 kHz, scan speed (V) at 297.19 mm/s, track displacement (TD) at 8 µm, and layer thickness (LT) at 1 µm.
  - For AlA, the optimized parameters are: Laser intensity (I) at 75.08%, pulse frequency (f) at 18.28 kHz, scan speed (V) at 400 mm/s, track displacement (TD) at 12 µm, and layer thickness (LT) at 3 µm.

## Figures and Tables

**Table 1:** Chemical composition of Titanium Alloy (TiA), Nickel Alloy (NiA), and Aluminum Alloy (AlA).

Materials	Elemental Composition															
Titanium Alloy; TiA	Al	V	Fe	O	C	N	Y	Ti								
	6.11	4.0	0.18	0.18	0.01	0.01	<0.005	Bal.								
Nickel Alloy; NiA	C	Mn	Fe	S	Si	Cu	Ni	Cr	Al	Ti	Co	Mo	Ta	B	Nb	P
	0.03	0.08	18.36	0.001	0.07	0.12	53.54	18.31	0.57	0.88	0.20	2.86	0.004	0.002	4.86	0.008
Aluminum Alloy; AlA	Si	Fe	Cu	Mn	Mg	Cr	Zn	Ti	Al							
	0.5	0.5	3.8–4.9	0.3–0.9	1.2–1.8	0.1	0.25	0.15	Bal.							

**Table 2:** Important properties of TiA, NiA, and AlA.

Property	Ti-6Al-4V [39,40,41,42,43]		AA 2024 [44,45,46,47,48]		Inconel 718 [49,50,51,52,53]		Unit
	Condition	Value	Condition	Value	Condition	Value	
Density (*ρ*)	-	4.43	-	2.7	-	8.9	g/cm^3^
Melting temperature (*T_m_*)	-	1604–1660	-	520	-	1260–1335	°C
Specific heat capacity (*Cp*)	*C_p_* = 0.176.*T* + 540	*T ≤ T_m_*: 830.4	*C_p_* = 0.557.*T* + 877.6	921–1172	-	430–673	J/kg °C
Thermal conductivity (*K*)	*K* = 0.0156.T + 7	*T ≤ T_m_*: 32.74	*K* = −0.125.*T* + 226	300<*T*<*T_m_*:164–220	23–1200 °C	10.6–29.6	W/m °C
Latent heat (*L*)	-	418.680	-	397	-	227	KJ/Kg
Dynamic viscosity (*µ*)	-	5.20 × 10^−3^	-	1.3 × 10^−3^	-	-	Ns/m^2^
Emissivity (*ε*)	>760–1100 °C	0.7–0.98	600–800 K	0.1–0.3	100–200 °C	0.24–0.33	-
Absorptivity@1.064 µm (*A*)	500–1400 °C	0.28–0.41	-	0.07	300–1700 K	0.00–0.55	-

**Table 3:** Variables and performance indicator.

Variables	Units	Levels			Performance Indicators	Units
Lamp current intensity (I)	A (%)	75	80	85	MRR_%_TiA_	µm^3^/s
Pulse frequency (f)	kHz	10	15	20	MRR_%_NiA_	µm^3^/s
Scanning speed (V)	mm/s	200	300	400	MRR_%_AiA_	µm^3^/s
Track displacement (TD)	µm	8	10	12		
Layer thickness (LT)	µm/scan	1	2	3		

**Table 4:** Response surface method, face centered central composite design (RSM FCCCD) and the selected experimental results after laser beam machining (LBM) of TiA, NiA and AlA.

Run #	Parameters					Responses						
	I (%)	F (kHz)	V (mm/s)	TD (µm)	LT (µm/scan)	MRR_th_ (µm^3^/s)	Titanium Alloy (TiA)		Nickel Alloy (NiA)		Aluminum Alloy (AlA)	
							MRR_act_TiA_ (µm^3^/s)	MRR_%_TiA_ (%)	MRR_act_NiA_ (µm^3^/s)	MRR_%_NiA_ (%)	MRR_act_AlA_ (µm^3^/s)	MRR_%_AlA_ (%)
1	75	20	200	12	1	24.00	18.00	75%	12.00	50%	0.00	0%
2	75	20	200	8	1	16.36	20.45	125%	10.91	67%	2.73	17%
3	80	15	300	10	2	66.67	33.33	50%	77.78	117%	183.33	275%
4	75	10	400	8	1	30.51	38.14	125%	45.76	150%	221.19	725%
5	80	15	300	10	2	66.67	33.33	50%	61.11	92%	211.11	317%
-	-	-	-	-	-	-	-	-	-	-	-	-
21	80	15	300	10	2	60.00	35.00	58%	65.00	108%	195.00	325%
22	85	20	400	8	1	30.51	55.93	183%	78.81	258%	203.39	667%
23	75	10	200	8	1	16.36	35.45	217%	46.36	283%	130.91	800%
24	85	20	400	12	1	46.15	57.69	125%	96.15	208%	273.08	592%
25	75	20	400	8	3	120.00	10.00	8%	20.00	17%	10.00	8%
-	-	-	-	-	-	-	-	-	-	-	-	-
41	75	15	300	10	2	66.67	22.22	33%	38.89	58%	105.56	158%
42	80	10	300	10	2	66.67	44.44	67%	88.89	133%	311.11	467%
43	80	15	300	10	2	66.67	33.33	50%	88.89	133%	177.78	267%
44	80	15	200	10	2	46.15	42.31	92%	53.85	117%	153.85	333%
45	80	15	300	12	2	78.26	39.13	50%	39.13	50%	234.78	300%

**Table 5:** ANOVA, Pareto and normal effects summary for TiA, NiA, and AlA.

Substrate Material	ANOVA Summary					Pareto and Normal Effects Summary			
	Significant Terms			Total Significant Terms	Top Most Significant Terms	Largest Effects		Moderate Effects	
	Linear Terms	Square Terms	Interaction Terms			Largest +ve Effect	Largest -ve Effect	Moderate +ve Effect	Moderate -ve Effect
TiA	5 terms I f V TD LT	1 term LT*LT	7 terms I*TD I*LT f*TD f*LT V*TD V*LT TD*LT	13 terms	3 terms LT I LT*LT	2 terms I LT*LT	1 term LT	5 terms V*LT TD*LT f*LT f*TD V*TD	5 terms I*LT V f TD I*TD
NiA	5 terms I f V TD LT	1 term LT*LT	6 terms I*f I*TD I*LT f*LT V*LT TD*LT	12 terms	2 terms LT I	1 term I	1 terms LT	5 terms LT*LT V*LT f*LT I*f TD*LT	5 terms f I*LT V D I*TD
AlA	5 terms I f V TD LT	2 terms I*I LT*LT	9 terms I*f I*V I*TD I*LT f*V f*TD f*LT V*LT TD*LT	16 terms	5 terms LT F I f*LT I*LT	2 terms I f*LT	3 terms LT F I*LT	6 terms f*V LT*LT f*TD TD*LT V*LT I*I	5 terms TD V I*V I*TD I*f

**Table 6:** Descriptive statistics: MRR%_TiA, MRR_%__NiA, MRR_%__AlA.

Variable	N	Mean	SE Mean	StDev	Median	Maximum	Skewness	Kurtosis
MRR%_TiA	54	85.5	10.8	79.0	50.0	383.3	1.93	3.66
MRR%_NiA	54	133.6	13.9	102.4	100.0	500.0	1.68	2.91
MRR%_AlA	54	399.4	49.9	366.3	300.0	1691.7	1.76	3.48

**Table 7:** Summary of the proposed models.

Response	S	R-sq	R-sq (adj)	R-sq (pred)
MRR%_TiA	10.93	98.55%	98.09%	96.90%
MRR%_NiA	31.40	92.72%	90.59%	84.94%
MRR%_AlA	43.54	99.01%	98.59%	97.38%

**Table 8:** Optimization goal for TiA, NiA and AlA.

Response	Goal	Lower	Target	Upper	Weight	Importance
MRR%_TiA	Target	0	100	383.333	1	1
MRR%_NiA	Target	8.33333	100	500	1	1
MRR%_AlA	Target	0	100	1691.67	1	1

**Table 9:** Multiple response optimal settings and optimization results for TiA, NiA, and AlA.

Variable	Optimal Settings			Optimization Results			
	TiA	NiA	AlA	Response	Fit	SE Fit	95% CI
I	80	75.54	75.08	MRR%_TiA	100.00	2.13	(95.70, 104.30)
f	15	19.57	18.28	MRR%_NiA	100.0	16.1	(67.4, 132.6)
V	300	297.19	400	MRR%_AlA	100.0	26.3	(46.7, 153.3)
TD	10	8	12				
LT	1.51	1	3				

**Table 10:** Multiple response prediction solutions for TiA, NiA and AlA.

Response	Sol.	I (%)	f (kHz)	V (mm/s)	TD (µm)	LT (µm)	MRR% Fit	Composite Desirability
MRR%_TiA	1	80.00	15.00	300.0	10.00	1.51	100.00	1.00
	2	84.69	20.00	400.00	12.00	1.14	104.51	0.98
	3	75.00	11.28	400.00	8.33	1.05	107.24	0.97
MRR%_NiA	1	75.54	19.57	297.19	8.00	1.00	100.00	1.00
	2	84.11	10.00	200.00	10.98	2.88	100.33	0.99
	3	75.32	10.32	211.33	8.12	2.53	101.04	0.99
MRR%_AlA	1	75.08	18.28	400.00	12.00	3.00	100.00	1.00
	2	75.10	10.08	400.00	12.00	3.00	142.73	0.97
	3	84.53	20.00	399.57	12.00	3.00	156.57	0.96
